# Model-based estimation of the state of vehicle automation as derived from the driver’s spontaneous visual strategies

**DOI:** 10.16910/jemr.12.3.10

**Published:** 2021-02-09

**Authors:** Damien Schnebelen, Camilo Charron, Franck Mars

**Affiliations:** Université de Nantes, CNRS, LS2N, France; University of Rennes 2, LS2N, France

**Keywords:** automated driving, manual driving, gaze behaviour, gaze dynamics, eye movement, region of interest

## Abstract

When manually steering a car, the driver’s visual perception of the driving scene and his or her motor actions to control the vehicle are closely linked. Since motor behaviour is no longer required in an automated vehicle, the sampling of the visual scene is affected. Autonomous driving typically results in less gaze being directed towards the road centre and a broader exploration of the driving scene, compared to manual driving. To examine the corollary of this situation, this study estimated the state of automation (manual or automated) on the basis of gaze behaviour. To do so, models based on partial least square regressions were computed by considering the gaze behaviour in multiple ways, using static indicators (percentage of time spent gazing at 13 areas of interests), dynamic indicators (transition matrices between areas) or both together. Analysis of the quality of predictions for the different models showed that the best result was obtained by considering both static and dynamic indicators. However, gaze dynamics played the most important role in distinguishing between manual and automated driving. This study may be relevant to the issue of driver monitoring in autonomous vehicles.

## Introduction

Driving is a complex dynamic task in which the driver must continuously process information from the environment. This is necessary to control the speed and direction of the vehicle; to gather information about other vehicles, road signs or potential hazards; and to make decisions about the route to be taken. Theoretical models of driving activity have been developed to account for this complexity. Michon, for example, proposed dividing driving activity into three hierarchically organised levels: the strategic level, the tactical level and the operational level (1). The strategic level corresponds to the definition of general driving goals, such as itinerary selection. At the tactical level, objects are recognised, danger is assessed and acquired rules are used to make short-term decisions. These decisions are implemented at the operational level, which is underpinned by online perceptual-motor loops.

During manual driving, drivers perform all the subtasks associated with the three control levels. However, during automated driving, some tasks are transferred to the automation. Then, the driver's role depends on the level of automation, as defined by the Society of Automotive Engineers (2). When lateral and longitudinal control are delegated to automation (SAE level 2), drivers become system supervisors rather than actors. This transformation of the driver's role changes their visual processing of the environment and their interaction with the vehicle (3).

At SAE Level 2, drivers are required to maintain their attention on the road so that they can regain manual control of the vehicle without delay at all times. However, the visuo-motor coordination necessary for the online control of steering and braking is no longer required, which constitutes a neutralization of the operational control loop. (4) showed that eye movements typically preceded steering-wheel movements by 800 ms in manual driving. Once control of the steering wheel is delegated to the automaton, this perceptual-motor coupling is no longer necessary. This explains why even in the absence of a secondary task, and with the instruction to monitor the driving scene, changes in gaze behaviour were observed under such conditions. Drivers who no longer have active control of the steering wheel tend to neglect short-term anticipation of the road ahead; they produce more distant fixations, known as “look-ahead fixations” (5, 6).

At SAE Level 3, drivers might no longer actively monitor the driving scene. When the system perceives that it can no longer provide autonomous driving, it issues a takeover request, allowing the driver a time budget to restore situational awareness and regain control of the vehicle. Failure to monitor the driving scene for an extended period is tantamount to neutralizing both the tactical control loop and the operational loop. During this time, drivers can engage in secondary tasks. The distraction generated by the secondary task results in less attention being directed towards the road than in the case of manual driving (7, 8, 9). One possible way to measure this shift of attention is to compute and compare the percentage of time spent in road centre (PRC; (10). Low PRC is associated with drivers being out of the operational control loop (8, 11). However, even without a secondary task, sampling of the driving environment is affected, with higher horizontal dispersion of gaze during automated driving compared to manual (12, 13, 14). All previous studies point to the same idea: the gaze is directed more towards the peripheral areas than the road during automated driving. However, observations based on the angular distribution of gaze only capture an overall effect and do not consider the gaze dynamics. For this purpose, a decomposition of the driving scene into areas of interest (AOIs) may be more appropriate. The decomposition into AOIs for the analysis of gaze while driving has been carried out in several studies (8, 11, 15), but only a few studies have examined the transitions between the different AOIs. Such a method was implemented by (16) to determine AOI fixation sequences as a function of driver experience and road type in manual driving. (17) similarly proposed analysing the fixation sequences during lane-change manoeuvres. The present study uses this approach to assess the driver’s gaze behaviour during an entire drive of either manual or automated driving.

Previous documented studies have examined the consequences of automated driving for drivers' visual strategies. For example, PRC has been shown to be low during automated rides. The present study uses the opposite methodological approach, aiming to determine the state of automation (i.e., manual or automated) based on the driver's spontaneous visual strategies. Of course, in a real driving situation, the state of automation of the vehicle is known and does not need to be estimated. Estimating the state of automation is therefore not an objective in itself, or at least not an objective with a direct applicative aim. Predicting the state of automation must be considered here as an original methodological approach that aims above all to identify the most critical oculometric indicators to estimate the consequences of automation on visual strategies. This methodology, if conclusive, could be useful for monitoring other driver states and understanding the underlying visual behaviour.

Driver visual strategies were considered in several ways. The analysis was performed by first considering only the PRC, and then a set of static indicators (percentage of time spent in the different AOIs) and/or dynamic indicators (transitions between AOIs). Partial Least Square (PLS) regressions were used to estimate a score between -1 (automated driving) and 1 (manual driving). This estimate of the state of automation from the visual indicators considered was used to classify the trials, with a calculation of the quality of the estimate in each case.

The objective was to determine whether considering the PRC was sufficient to discriminate between automated and manual driving. The extent to which the inclusion of other static and dynamic indicators would improve the driver-state estimation was also assessed. We hypothesised that considering the gaze dynamics would improve the ability to distinguish between manual and automated driving.

## Methods

### Participants

The study sample included 12 participants (9 male; 3 female) who had a mean age of 21.4 years (SD = 5.34). They all had normal or corrected vision (with contact lenses only). All participants held a French driver’s licence, with mean driving experience of 9950 km/year (SD = 5500). A signed written informed consent form, sent by e-mail one week before the experiment and printed for the day of experimentation, was required to participate in the study.

### Materials

**Figure 1:**
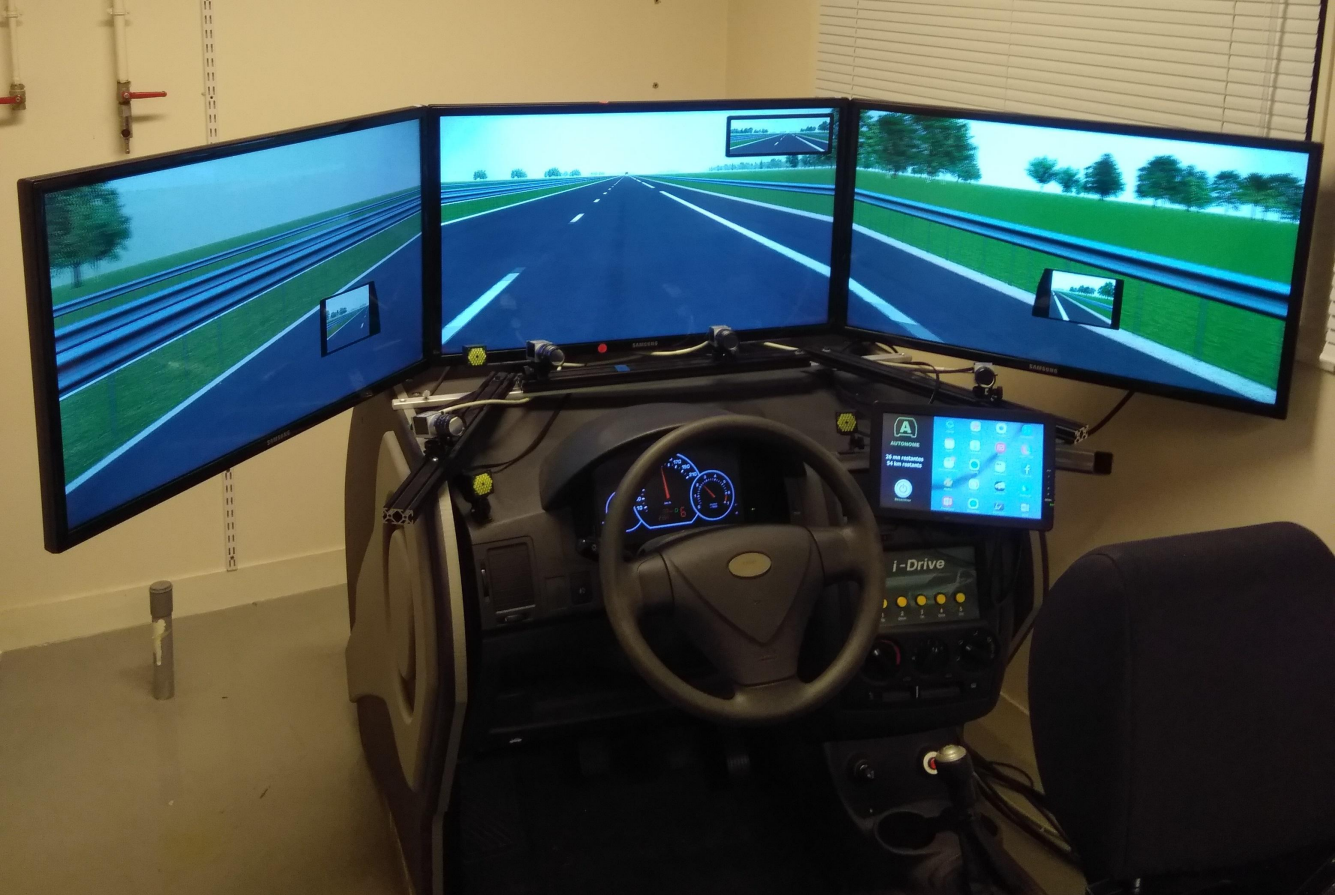
Driving simulator environment

The study used a fixed-base simulator consisting of an adjustable seat, a digital dashboard, a steering wheel with force feedback, a gear lever, a clutch, an accelerator and brake pedals (see figure 1). The driving scene was generated with SCANeR Studio (v1.6) and displayed on three large screens in front of the driver (field of view ~= 120°). An additional screen, simulating a central console, provided information about the vehicle automation mode.

Gaze data were recorded using a Smart Eye Pro (V5.9) eye-tracker with four cameras (two below the central screen and one below each peripheral screen). The calibration of the eye-tracker occurred at the start of the experiment and required two steps. In the first step, a 3D model of the driver’s head was computed. In the second step, the gaze was computed using a 12-point procedure. The overall accuracy of calibration was 1.2° for the central screen and 2.1° for peripheral areas. Gaze data and vehicle data were directly synchronised at 20 Hz by the driving simulator software.

### Procedure

After adjusting the seat and performing the eye-tracker calibration, participants were familiarised with the simulator by driving manually along a training track. Once this task was completed, instructions for automated driving were given orally. These were as follows: when the autonomous mode was activated, vehicle speed and position on the road would be automatically controlled, taking into account traffic, speed limits and overtaking other cars if necessary. To fit with SAE level 3 requirements, drivers were told that the automated function would only be available for a portion of the road, with the distance and time remaining in the autonomous mode displayed on the left side of the human-machine interface (HMI). When automation conditions were not met, the system would request the driver to regain control of the vehicle.

**Figure 2:**
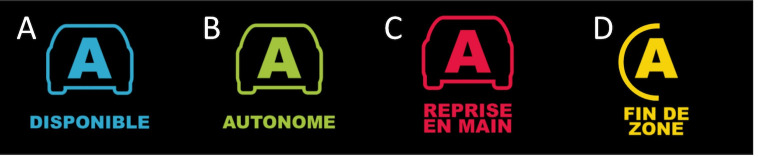
Pictograms displayed on the HMI. A: autonomous driving available; B: autonomous driving activated; C: take-over request (8 s); D: take-over request (45 s)

Two use cases were presented to the participants. In the first case, the vehicle was approaching the end of the automated road section. The drivers received mild auditory and visual warning signals and had 45 s to regain control. The second use case was an unexpected event, such as the loss of sensors. In this case, a more intense auditory alarm and a different pictogram were displayed, and drivers had only 8 s to resume control. All the pictograms (figure 2) and sounds used by the HMI were presented to participants at the end, before they started a second training session.

During the second training session, participants first experienced manual driving (with cruise control, corresponding to SAE level 1) and then automated driving (SAE level 3: conditional automation). At level 3, drivers experienced four transitions from automated to manual driving, two in each use case presented in the instructions. All takeovers were properly carried out during the training session.

Then, after a short break, the experimental phase started. All participants experienced the manual and automated conditions and the order of presentation was counter-balanced. The scenario was similar in the two driving conditions and comprised an 18-min drive in a highway context. Most of the road was a 40-km two-lane dual carriageway, with a speed limit of 130 km/h, in accordance with French regulations. Occasional changes in road geometry (temporary three-lane traffic flow, highway exits and slope variation) and speed limits (130 km/h to 110 km/h) were included to make the driving less monotonous. In both directions on the highway, traffic was fluid, with eight overtaking situations.

A critical incident occurred at the 18th minute of automated driving. Thereafter, a questionnaire was administered. However, these results are not reported as they are beyond the scope of this paper, which merely aims to characterise differences in gaze behaviour for manual versus automated conditions. Therefore, only the data common to both conditions is considered here; that is, 17 min of driving time, during which no major events occurred.

### Data Structure and Annotations

**Figure 3:**
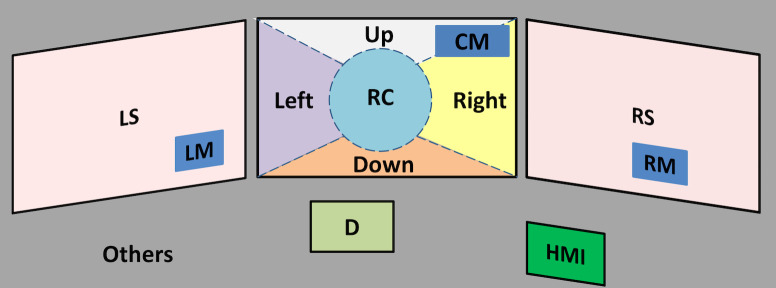
Division of the driving environment in 13 areas of interest

The driving environment was divided into 13 AOIs as shown in figure 3. These were as follows:

- The central screen contained six areas: the central mirror (area CM); the road centre (RC), defined as a circular area of 8° radius in front of the driver (10); and four additional areas defined relative to the road centre (Up, Left, Down and Right; see (8)).

- Each peripheral screen contained two areas: the lateral mirror (LM, RM) and the remaining peripheral scene (LS, RS).

- The dashboard (D).

- The HMI.

- All gaze data directed outside of the stipulated areas were grouped as an area called “Others”.

The percentage of time spent in each AOI was computed, as was a matrix of transitions between AOIs. The transition matrix indicated the probability to shift from one AOI to another or to remain in the same AOI. Probabilities were estimated by the observations made on the participants. As there were 13 AOIs that defined the entire world, the transition matrix was a 13*13 matrix.

24 statistical units were considered (= 12 participants * 2 driving conditions). For each of them, 182 visual indicators (= 13*13 transitions + 13 percentage of time on each AOI) were calculated, forming a complete set of data including both static and dynamic gaze indicators. The complete matrix was therefore 24*182 and was denoted as **X_DS_**. When only the transitions were considered, the matrix was denoted as **X_D_** and its size was 24*169. When only the percentages of time spent on each AOI were considered, the matrix was 24*13 and was denoted as **X_S_**. Another vector, constituted of only the PRC, was also computed (size: 24*1) and was labelled **X_PRC_**.

All the matrices were centred and reduced for the next step of the analysis. As described below, this entailed PLS regressions.

The data were structured according to the aim of predicting the automation state (**Y**) from either **X_PRC_**, **X_S_**, **X_D_** or **X_DS_**. The automation state was defined as follows: it was valued 1 for manual driving and -1 for automated driving.

The appropriate model of prediction was chosen with respect to certain constraints. First, the number of visual indicators (max 182 for **X_DS_**) available to explain **Y** was notably higher than the number of observations (n=24). Second, the visual indicators were correlated. Indeed, with the driving environment divided into 13 AOIs, the percentage of time spent on 12 AOIs enabled calculating the percentage of time spent on the 13th AOI. In mathematical terms, **X** might not be full rank. Given this correlation between variables, a simple linear model was not appropriate.

Considering these constraints, the PLS regression model was selected. PLS regression yields the best estimation of **Y** available with a linear model given the matrix **X** (18). All PLS regressions performed in this study used the PLS regression package on R (19).

PLS regression is based on a simultaneous decomposition of both **X** and **Y** on orthogonal components. Once the optimal decomposition is found, the number of visual indicators is systematically reduced to consider only relevant visual indicators for the prediction. Ultimately, the most accurate model – involving optimal decomposition and only the most relevant visual indicators – is set up to provide the estimation of **Y**, based on a linear combination of the relevant visual indicators. Validation was performed using a leave-one-out procedure. For more details, see the Appendix section.

### Data Analysis

Three sequential stages composed the analysis:

- The first step only considered the percentage of time spent on each AOI individually. The difference between manual and automated driving was evaluated using paired t-tests, corrected with the Holm-Bonferroni procedure to control the family-wise error rate.

- In the second step, prediction models of the automation state were developed using PLS regression. Several models were developed, depending on the nature of visual indicators that served as input: PRC only (**X_PRC_**), static indicators only (**X_S_**), dynamic indicators (**X_D_**) or a combination of static and dynamic indicators (**X_DS_**).

- In the final step, a binary classification of the prediction was performed. A given statistical individual was classified as “automated” if its prediction was negative and as “manual” in the opposite case. The number of errors was then considered.

## Results

### Distribution of visual attention as a function of the automation state

The percentage of time spent in each of the 13 AOIs during the entire drive is presented in table 1.

**Table 1:** Distribution of visual attention for manual and automated drives.

Percentage of time spent on each AOI	Manual Driving	Automated Driving	Auto - Manu Difference
Road Centre	67.45	40.76	-26.69
Right Mirror	0.01	0.07	0.06
Left Mirror	0.137	0.31	0.173
HMI	0.01	0.31	0.3
Down Area	0.32	0.67	0.35
Left Area	1.561	2.09	0.529
Right Area	2.91	4.46	1.55
Dashboard	2.08	4.45	2.37
Central Mirror	4.084	6.9	2.816
Up Area	14.74	17.99	3.25
Right Screen	2.679	6.78	4.101
Left Screen	2.6	7.14	4.54
Others Area	1.48	8.03	6.55

During manual driving, participants spent about 27% more time in the Road Centre area than during automated driving (p<.001). Conversely, automated driving was associated with more gazing directed all other areas, in particular to the left and right peripheral screens, the down area and the central mirror, although these differences failed to reach statistical significance (p<.10).

### Predictions of the automated state with PLS regression

**Figure 4:**
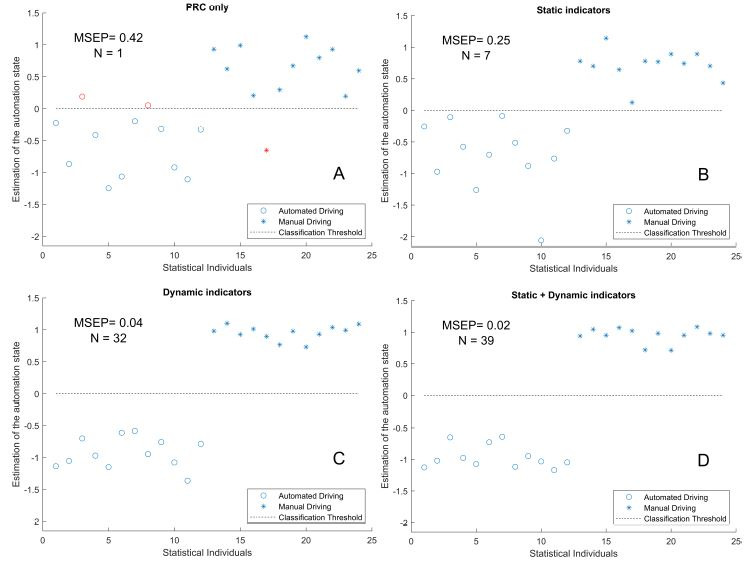
Estimations (markers) of the automation state from: A, PRC only; B, static indicators only; C, dynamic indicators only; D, static and dynamic indicators. The marker type indicates the real automation state: circle for automated driving, star for manual driving. The MSEP and number of visual indicators retained during the PLS process (N) are annotated in text. Errors of classifications (relative to the classification threshold in black) are indicated in red.

Several predictions were performed with PLS predictions, depending on the input matrix. These were as follows: **X_PRC_** (PRC only), **X_S_** (static indicators only), **X_D_** (dynamic indicators) or the combination of dynamic and static indicators (**X_DS_**). Estimations obtained with the PLS regression method are presented in figure 4.

Results showed that considering PRC alone provided a rough estimation of the automation state, with a mean square error of prediction (MSEP) of 0.42. It gave rise to three classification errors (figure 4-A). When all static indicators were considered as input, seven were selected for PLS regression (table 2, column 1). This analysis yielded a more accurate estimation of the automation state than prediction based on PRC alone; the MSEP was halved and there was no classification error. However, four statistical individuals (1, 3, 8 and 17) remained close to the classification threshold (figure 4-B).

When dynamic indicators alone were considered, 32 (out of 169) indicators were selected by the PLS regression. The prediction results were greatly improved, with an MSEP of 0.04. Moreover, the manual and automated driving conditions could be more clearly discriminated (figure 4-C). Considering static and dynamic indicators together yielded the lowest prediction error (MSEP = 0.02), although the pattern of results was quite similar to those obtained for dynamic PLS (figure 4-D). It should be noted that the indicators selected by the combined static and dynamic PLS were the same as those selected for static PLS and for dynamic PLS.

The PLS regression coefficients for each visual indicator retained in the different predictions are presented in table 2. The sign of the coefficients indicates the direction of their contribution to the estimate. A positive coefficient tends to increase the estimated score and is therefore a feature of manual driving. A negative coefficient decreases the estimated score, which corresponds to automated driving. A coefficient’s absolute value indicates its importance for the prediction, with large coefficients contributing strongly to the estimation.

**Table 2:** PLS regression coefficients per visual indicator. A positive coefficient corresponds to a visual indicator characteristic of manual driving. A negative coefficient is associated with automated driving

Visual Indicator	Static	Dynamic	Static Dynamic
Percentage of time spent in the Road Centre	0.257		0.058
Percentage of time spent in the Dashboard Area	-0.149		-0.034
Percentage of time spent in the Others Area	-0.171		-0.039
Percentage of time spent in the Left Screen	-0.191		-0.043
Percentage of time spent in the Right Screen	-0.194		-0.044
Percentage of time spent on the Central Mirror	-0.203		-0.046
Percentage of time spent in the Down Area	-0.202		-0.046
Transition from the Left Area to the Road Centre		0.064	0.051
Transition from the Up Area to the Road Centre		0.058	0.047
Transition from the Right Screen to the Road Centre		0.057	0.045
Transition from the Down Area to the Road Centre		0.055	0.044
Transition from the Right Area to the Road Centre		0.052	0.042
Transition from the Left Screen to the Road Centre		0.052	0.041
Transition from the Dashboard Area to the Left Screen		0.048	0.039
Transition from the Right Mirror to the Left Mirror		0.046	0.037
Transition from the Right Screen to the Down Area		0.046	0.037
Transition from the Others Area to the Road Centre		0.046	0.037
Transition from the Road Centre to the Down Area		0.045	0.036
Transition from the HMI to the Central Mirror		0.044	0.035
Transition from the Dashboard to the Road Centre		0.041	0.033
Transition from the Others Area to the Dashboard		0.042	0.033
Transition from the HMI to the Others Area		-0.033	-0.025
Transition from the Right Area to the Dashboard		-0.040	-0.032
Transition from the Central Mirror to the Dashboard		-0.040	-0.032
Transition from the Down Area to the HMI		-0.044	-0.035
Transition from the Left Area to the Central Mirror		-0.046	-0.036
Transition from the Road Centre to the HMI		-0.046	-0.037
Transition from the Central Mirror to the Left Mirror		-0.047	-0.037
Transition from the Others Area to the Others Area		-0.046	-0.037
Transition from the Up Area to the Up Area		-0.048	-0.039
Transition from the Road Centre to the Others Area		-0.049	-0.039
Transition from the Down Area to the Down Area		-0.052	-0.042
Transition from the Central Mirror to the Right Screen		-0.056	-0.044
Transition from the Others Area to the Left Screen		-0.057	-0.044
Transition from the Left Area to the Right Area		-0.059	-0.047
Transition from the Left Area to the Left Area		-0.057	-0.05
Transition from the Down Area to the Central Mirror		-0.067	-0.052
Transition from the Road Centre to the Central Mirror		-0.069	-0.055
Transition from the HMI to the HMI		-0.081	-0.065

In accordance with the analysis presented in table 1, the only static indicator selected by the PLS analyses as a feature of manual driving was the percentage of time spent looking at road centre. By contrast, automated driving was characterised by a combination of looking at the peripheral areas (left and right screen), the dashboard, Others, central mirror and the down area.

**Figure 5:**
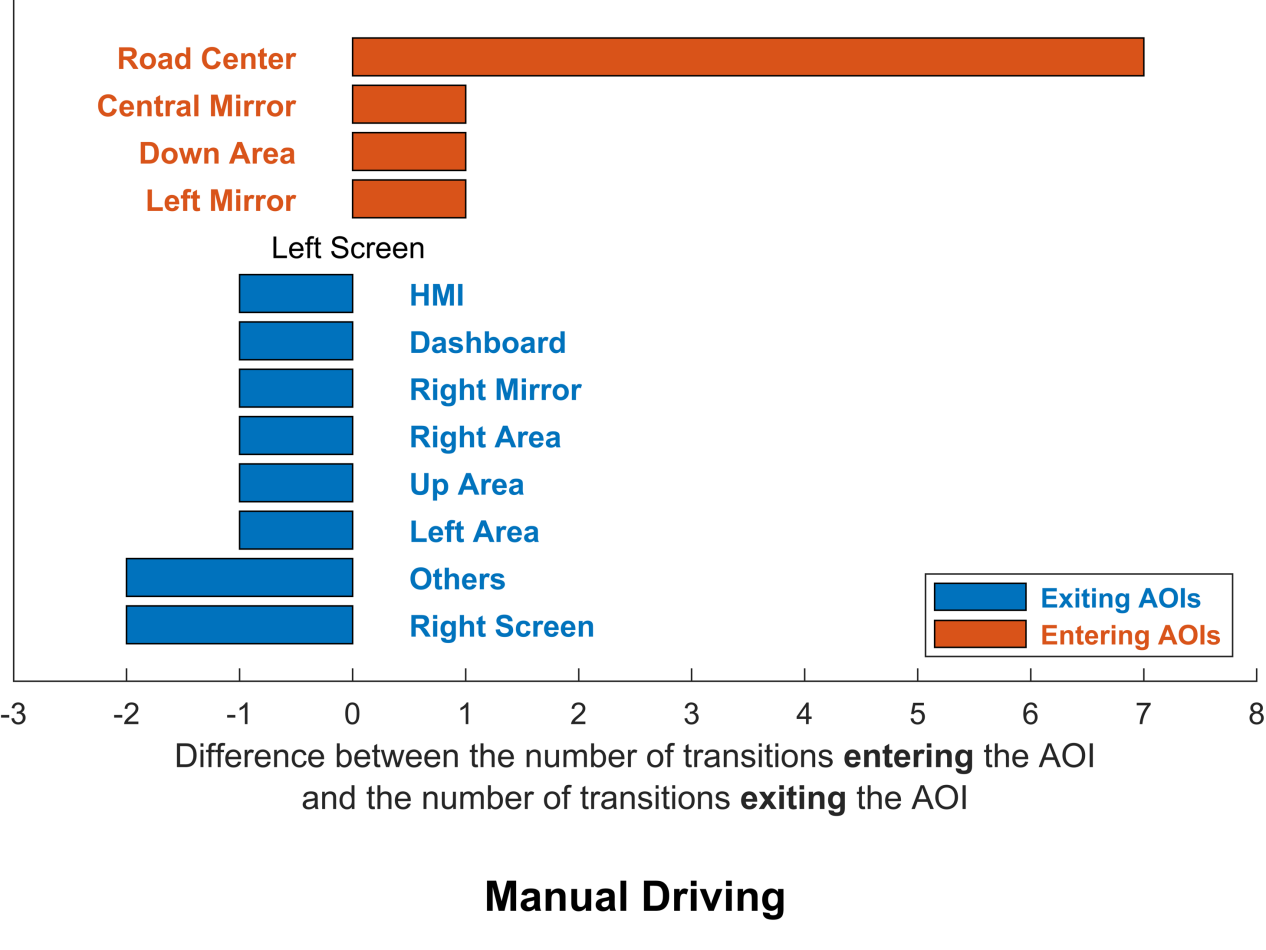
Visualisation of exiting AOIs (blue) and AOIs (orange) for manual driving

**Figure 6:**
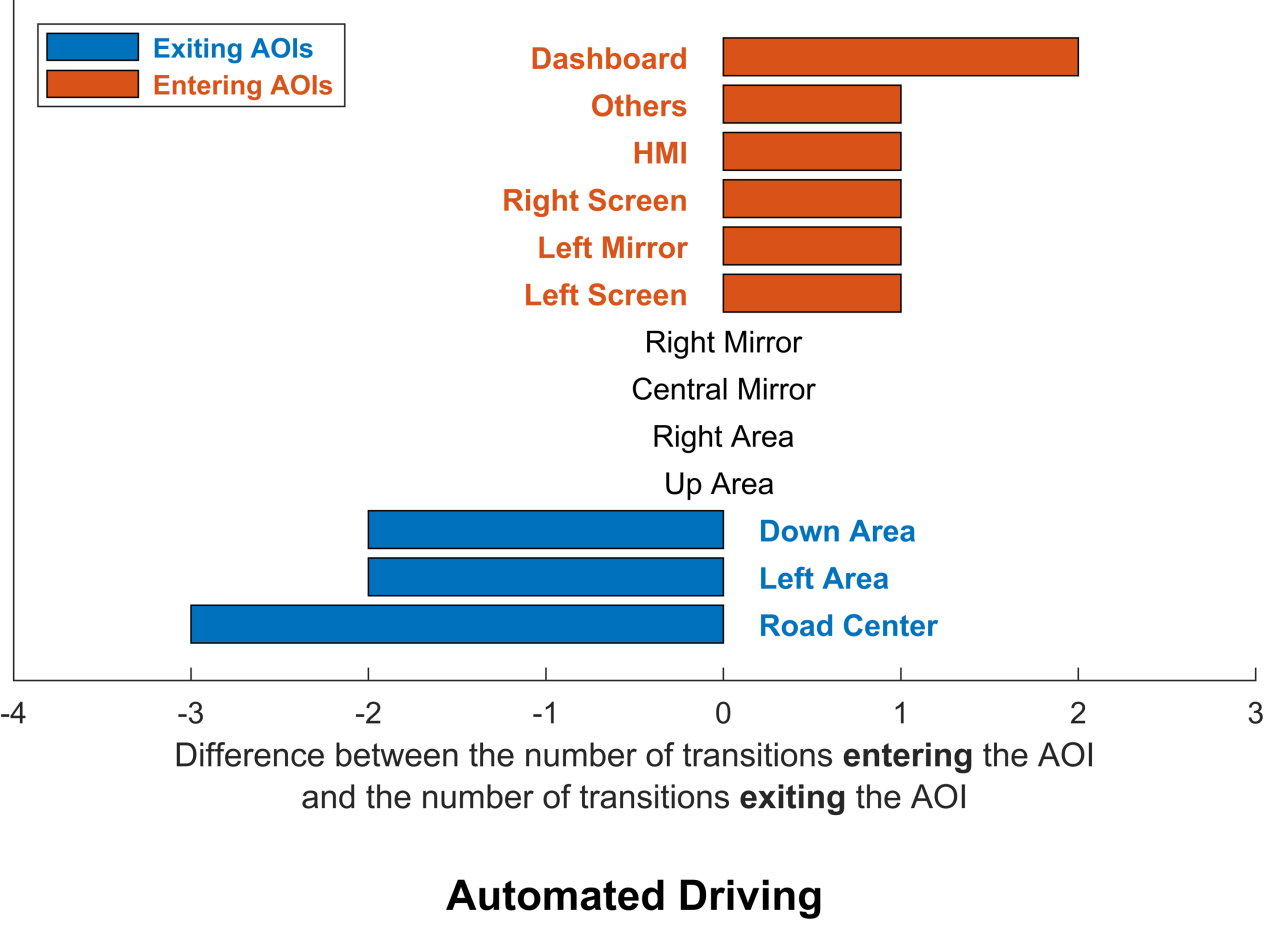
Visualisation of exiting AOIs (blue) and entering AOIs (orange) for automated driving.

Both dynamic PLS and the combined static and dynamic PLS selected 32 AOI transitions, 14 related to manual driving and 18 related to automated driving. To facilitate the identification of dynamic visual patterns characteristic of manual or automated driving, we performed a final analysis of the AOI transitions. We examined whether the gaze mostly entered or exited each AOI. The difference between the number of transitions entering the AOI and the number of transitions exiting the AOI was calculated. If the resulting value was positive, the AOI was classed as an entering AOI; if the value was negative, the AOI was classed as an existing AOI. The results are presented in figures 5 and 6.

The analysis of gaze dynamics during manual driving showed that many more glances were coming in than going out the road centre area. To a lesser extent, the area just below (down area) and the left and central mirror also received more glances in than out. All other AOIs had more exiting glances, apart from the left screen, which was at equilibrium.

Automated driving was characterised by many glances moving away from the road (road centre, left area and down area), and a favouring of areas that provided information about the vehicle’s status (dashboard, HMI) and the adjacent lane (left mirror, left screen). More glances entered the areas not related to the driving task (Others, right screen) than exited.

## Discussion

The aim of this work was to try to predict, on the basis of drivers' visual strategies, whether they were driving manually or in autonomous mode. This estimation allowed the identification of the most important visual indicators in both driving modes. To do this, we analysed how drivers distributed their attention over a set of AOIs that composed the driving environment. We considered both static indicators (percentage of time spent on AOIs) and indicators of gaze dynamics (matrices of transitions between AOIs). The indicators that best predicted the driving mode were selected using PLS regression. The quality of the prediction was then evaluated by examining the ability of the models to distinguish between the two driving modes without error.

The results confirm a recurrent observation in studies of visual strategies in autonomous driving situations. That is, drivers spend less time looking at the road and more time looking at the periphery (7, 12, 13, 14). Under the experimental conditions in this study, the diversion of the gaze from the road centre area was partly in favour of the lateral visual scene, the central rear-view mirror, the dashboard and the down area. The results can be interpreted in relation to the concept of situation awareness (20). Situational awareness is built at three levels. First, drivers must perceive (level 1, perception) and understand visual information (level 2, comprehension). Drivers must also anticipate future driving situations (level 3, projection). During automated driving, in the absence of strong sources of distraction, drivers were freed from vehicle control and could therefore take information about the driving environment and the state of the vehicle at leisure. The dispersed gaze observed could thus have contributed to maintaining better situational awareness. However, an increase in the number of glances directed at places not relevant to the driving task (Other area) was also reported. Hence, even in the absence of a major source of distraction or secondary task, some disengagement was observed.

In comparison, manual driving appeared stereotypical, with more than two thirds of the driving time spent looking at the road ahead. There were also many transitions back to this area once the gaze had been turned away from it. During manual driving, visuomotor coordination is essential: gaze allows the anticipation of the future path and leads steering actions (4, 21, 22). It has been shown that autonomous driving makes visuomotor coordination inoperative, which can have deleterious consequences in the event of an unplanned takeover request (3, 5, 6, 23).

Previous work has shown that automation of driving is associated with reduced PRC (8, 11). Here, we examined the corollary by attempting to predict the state of driving automation based on the observed PRC. The results showed that PRC was indeed a relevant indicator. Categorising drivers on this criterion showed fairly accurate results, although with some error. By considering all areas of potential interest in driving, the quality of prediction increased significantly. The improvement in prediction notably depended on the nature of the indicators. Although it was possible to categorise – without error – the mode of driving according to the percentage of time spent on all AOIs, the quality of prediction was far higher when we also considered the dynamics of transitions between AOIs. The MSEP was reduced 6-fold using the dynamic approach compared to the static approach. Moreover, adding static indicators to dynamic indicators yielded the same pattern of results as that obtained from dynamic indicators alone, with slightly less prediction error.

These results confirm the hypothesis that gaze dynamics are strongly impacted by automation of the vehicle. Specifically, gaze dynamics appear to explain the essence of the distinction between manual and autonomous driving.

As mentioned, considering the transitions between AOIs improved the prediction of the driving mode. It also enabled refining the visual patterns characteristic of manual or automated driving. For example, during manual driving, static indicators highlighted the predominance of information obtained from the road centre area. Accounting for the gaze dynamics confirmed this observation but also revealed a subtle imbalance in terms of reception versus emission for the left and central rear-view mirrors. Even if glances at the mirrors do not account for substantial visual processing time, they might be essential for the driver to create a mental model of the driving environment.

This study developed a methodology to analyse the influence of the level of automation based on a large set of visual indicators. This methodology is based on PLS regression. Other modeling approaches, such as Long Short-Term Memory (LSTM) networks or Hidden Markov Models (HMMs), could lead to comparable results in terms of prediction quality. However, non-parametric approaches such as those using neural networks might prove less useful to achieve the objective of our study, which was to determine the most relevant visual indicators to discriminate driving conditions. This problem of interpretation may not arise with HMMs, which are quite common in the analysis of visual exploration of natural scenes (24). Nevertheless, to train an HMM requires to set prior probabilities, which is not the case with PLS regression.

The approach developed in this study could potentially be used to detect other driver states. For example, it could be a useful tool for detecting driver activity during automated conditional driving (25). In the event of a takeover request, the method could also be used to assess driver readiness (26). During autonomous driving, the driver may also gradually get out of the loop, which may lead to a decrease in the quality of takeover when it is requested (27). Recently, we have shown that PLS modelling can discriminate between drivers who supervise the driving scene correctly and drivers who are out of the loop with a high level of mind wandering (28). The approach could also be transposed to other areas such as the evaluation of the out-of-the-loop phenomenon in aeronautics (29).

## Conclusions

This study predicted a person’s driving mode from visual strategies, using PLS regression. The quality of this prediction depended essentially on gaze dynamics, although the optimal prediction was achieved by examining a combination of static and dynamic characteristics. This study helps to pave the way for developing algorithms to estimate the driver's state in an autonomous vehicle based on oculometric data (17, 30). Driver monitoring is a challenge in the development of new generations of these vehicles as it may be essential to assess whether the driver is in the loop or out of it, in terms of vehicle control and environmental supervision (13).

### Ethics and Conflict of Interest

The experiment was approved by the INSERM ethics committee (IRB00003888 and FWA00005831). All participants gave written informed consent in accordance with the Declaration of Helsinki. The authors declare that the research was conducted in the absence of any commercial or financial relationships that could be construed as a potential conflict of interest.

### Acknowledgements

This research was supported the French National Research Agency (Agence Nationale de la Recherche, AUTOCONDUCT project, grant n° ANR-16-CE22-0007-05). The authors are thankful to the participants for their voluntary participation to this experiment.

## Appendix

This appendix develops step-by-step the procedure to predict the automation state (**Y**) from a matrix of gaze behaviour (**X**) using PLS regressions. In the experiment, four matrixes of gaze behaviour were considered, so this procedure was applied four times.

**Step A: Calculating the optimal number of components**

Principle: PLS models are based on several orthogonal components, which constitute the underlying structure of the prediction model. With many components, the model is complex and highly accurate but also highly specific to the data. By contrast, few components mean a simpler model structure; the model may lose accuracy but can be more generalizable to other datasets. Thus, an optimal compromise in the number of components can prevent data overfitting while maintaining high accuracy.

Application: This compromise was sought by testing several numbers of components – from one to 10 components. The optimal number would minimise the mean square error of prediction (MSEP) with a leave-one-out procedure.

**Step B: Reducing the number of visual indicators**

In the previous step, an optimal structure of the prediction model was found, considering certain visual indicators (depending on the matrix of gaze behaviour) to predict the automation state. The aim of this next step was to increase the predictive power – that is, the percentage of variance in **Y** that was explained – by selecting fewer indicators that were relevant for the prediction.

Principle: The PLS regression is a linear statistical model. Therefore, the relationship between the input matrix (**X**) and the dependent variable to estimate $\hat{𝐘}$ was linear:

$$\hat{𝐘}=\;𝐗*𝐂$$

where **C** is the matrix of the regression coefficients.

Coefficients can be interpreted as follows:

- Coefficient signs indicate the direction in which a visual indicator (from X) influenced the estimation of the automation state ($\hat{𝐘}$). If positive, the score increased, meaning that this indicator featured mainly in manual driving. By contrast, a negative coefficient meant that this indicator featured mainly in automated driving.
- A coefficient’s magnitude (absolute value) indicates the relative importance of each indicator. If the magnitude of a coefficient was close to zero, the contribution of the visual indicator to the prediction was negligible. By contrast, a large magnitude indicated a crucial indicator in the prediction.

Application: To reduce the number of visual indicators of **X**, coefficient magnitudes were compared with an increasing threshold value, which ranged from 0.01 to 0.2. A new PLS regression was computed for each partial matrix – that is, a matrix comprising only the indicators whose coefficient magnitude exceeded the threshold value. The threshold was increased by steps of 0.005 until the percentage of variance in **Y** that was explained by the partial model no longer increased.

At the end of this step, a partial matrix of gaze behaviour that included only the selected visual indicators was computed. This partial matrix was denoted ${𝐗}^{𝐩}$.

Step C: Computing the mean square error of prediction

The previous steps found the most appropriate parameters for PLS regression models. A new model that considered those parameters was set up, with its coefficients denoted ${𝐂}^{𝐩}$. Then, the estimations ($\hat{{𝐘}^{𝐩}}$**)** and the MSEP were calculated as follows:

$$\left\{ \begin{array}{c} \hat{{𝐘}^{𝐩}}={𝐗}^{𝐩}*{𝐂}^{𝐩}\\ \;MSEP\;=\bar{{\left(𝐘-\hat{{𝐘}^{𝐩}}\right)}^{2}}\; \end{array}\right.\;$$
